# Energy and Exergy Evaluation of a Two-Stage Axial Vapour Compressor on the LNG Carrier

**DOI:** 10.3390/e22010115

**Published:** 2020-01-17

**Authors:** Igor Poljak, Josip Orović, Vedran Mrzljak, Dean Bernečić

**Affiliations:** 1 Maritime Department, University of Zadar, Mihovila Pavlinovića 1, 23000 Zadar, Croatia; jorovic@unizd.hr; 2 Faculty of Engineering, University of Rijeka, Vukovarska 58, 51000 Rijeka, Croatia; vmrzljak@riteh.hr; 3 Faculty of Maritime Studies, University of Rijeka, Studentska 2, 51000 Rijeka, Croatia; bernecic@pfri.hr

**Keywords:** two-stage LNG compressor, energy losses, exergy destruction, energy efficiency, exergy efficiency

## Abstract

Data from a two-stage axial vapor cryogenic compressor on the dual-fuel diesel–electric (DFDE) liquefied natural gas (LNG) carrier were measured and analyzed to investigate compressor energy and exergy efficiency in real exploitation conditions. The running parameters of the two-stage compressor were collected while changing the main propeller shafts rpm. As the compressor supply of vaporized gas to the main engines increases, so does the load and rpm in propulsion electric motors, and vice versa. The results show that when the main engine load varied from 46 to 56 rpm at main propulsion shafts increased mass flow rate of vaporized LNG at a two-stage compressor has an influence on compressor performance. Compressor average energy efficiency is around 50%, while the exergy efficiency of the compressor is significantly lower in all measured ranges and on average is around 34%. The change in the ambient temperature from 0 to 50 °C also influences the compressor’s exergy efficiency. Higher exergy efficiency is achieved at lower ambient temperatures. As temperature increases, overall compressor exergy efficiency decreases by about 7% on average over the whole analyzed range. The proposed new concept of energy-saving and increasing the compressor efficiency based on pre-cooling of the compressor second stage is also analyzed. The temperature at the second stage was varied in the range from 0 to −50 °C, which results in power savings up to 26 kW for optimal running regimes.

## 1. Introduction

The harmful pollutant emissions significantly increase over the last years in industry, marine and other energy sectors [1]. Such an increase has many negative impacts on human health as well as on the marine environment [2].

In the last years, liquefied natural gas (LNG) is recognized by many scientists and experts as a good alternative to conventional fuels due to many reasons. On one side, from the pollution point of view, the combustion of LNG removes sulfur oxides (SO_X_) and particulate matter (PM) emissions almost completely, while at the same time carbon dioxide (CO_2_) and nitrogen oxides (NO_X_) can be significantly reduced [3,4]. On the other side, due to its low temperature (around −162 °C), LNG can be used as a heat sink in many power plants or industry processes [5,6,7] or its cold energy can be utilized in other applications [8]. Therefore, the usage of LNG can be beneficial in several different ways.

In the marine sector, most LNG carriers use boil-off gas (BOG) from the cargo tanks for its propulsion [9]. The domination of steam propulsion systems, which are traditionally applied on the LNG carriers [10,11] is nowadays highly influenced by dual-fuel diesel engines (DFDE) [12,13] and its possible upgrades [14]. Such dual-fuel diesel engines require a compressor unit which delivers LNG vapors from the cargo tanks to the engine.

It is very rare to find in the literature efficiency evaluation of this kind of compressor. The most scientific literature which deals with simulation and modeling of LNG systems is taking LNG (or other gas or vapor) compressor efficiency on presumptions according to the manufacturer specifications. A literature overview of several compressors is presented in Table 1, however, the type of each compressor is not known.

In some researches which deals with the LNG vapor compression, the authors clearly notify the type of compressor. As for an example, Reddy et al. [20] investigate reciprocating compressors from the LNG terminals and concluded that such compressors are notable energy consumers, which require proper optimization techniques, while Park et al. [21] analyzed dual-opposed linear compressor used in LNG (re)liquefaction process and its optimal compressor operating regimes.

In other researches, the authors investigate entire processes with LNG vapors, therefore, exact compressor types are not mentioned. As for an example, overall compression power reduction and exergy loss analysis for a cascade LNG process presented Nawaz et al. [22] where the authors found possibilities for minimization of the entire process exergy losses. Investigation of the LNG fuel gas supply system, where the compressors are used for various purposes, presented Park et al. [23]. Also in this research, the authors made several variations of the entire LNG fuel gas supply system, but the BOG compressor (or more of them) type is not presented.

The investigated DFDE LNG carrier has an electricity generating set which consists of four identical engine units. Each unit is designed to develop a total power of 7800 kW. Engines may consume diesel oil, heavy fuel oil, and LNG. LNG consuming requires pilot diesel oil burning to initiate the combustion process in LNG operation mode [24,25]. Gasified LNG is taken from the cargo tanks to the inlet of the two-stage compressor unit and delivered to the engines for completion and continuation of the combustion process [25,26]. As mentioned before, the burning of natural gas in the engines is a useful concept for reducing NO_X_ emissions according to Marpol Annex VI Tier 1 to Tier 3 requirements [27,28] but also it is accommodating request of maintaining cargo tank pressure in the required range, which is up to 25 kPa [29], although independent tanks class B may withstand pressures as high as 70 kPa [30]. As exploitation characteristics of this type of compressor, especially during on-board performance are rarely investigated, this paper will cover energy and exergy analysis of two-stage LNG compressor in real working conditions to better understand the power losses. Measurements were taken from the minimum flow request of the compressor, which is set from the manufacturer specifications. The engine load was gradually increased until the point of normal continuous rating at main propulsion shafts, which equals 56 rpm at each propulsion shaft.

## 2. Description and Characteristics of the Analyzed Two-Stage Compressor

Two-stage axial LNG compressor on LNG carrier is taking evaporated vapor from the cargo tanks, which varies in temperature at compressor inlet from about −130 to −60 °C. LNG vapor is pressurized inside the two stages to 650 kPa and delivered to the engines at a set pressure of 580 kPa [31]. Compressor capacity is controlled by variable diffuser vanes, which are adjusted according to header set pressure [31]. The temperature at the inlet of the compressor is desirable to be below −110 °C to avoid the operation of the compressor in the surge area. As previously mentioned, the suction temperature in the tank varies due to the delivery of compressor, weather conditions and pressure inside the tank. To achieve the target temperature of minimum −110 °C at the compressor inlet, the cargo spray pump injects a certain amount of liquid LNG into the suction vapor header line and in such way inlet temperature at the compressor’s first stage is maintained inside a required range. The maximum electromotor rating at full speed is 610 kW and at half speed is 85 kW. These two ratings determine rotation speed which may be selected as 3550 or 1775 rpm at the electromotor side. The lower selected motor power is used only for the gradual cooling of the compressor in the recirculation loop until the targeted temperature at compressor inlet, which is below −100 °C, is reached. Once the targeted temperature is achieved compressor has permission to start at a higher speed. Half speed is not in use in normal navigation mode, due to the low delivery pressure of the compressor to the engines and this option is useless for this type of engine. However, half a rotation speed option may be used for burning excess boil-off gas from cargo tanks in the gas combustion unit. The compressor is driven by an electromotor which is coupled to spur reduction gear. Particulars of the two-stage compressor are given in Table 2 [32].

**Figure 1:**
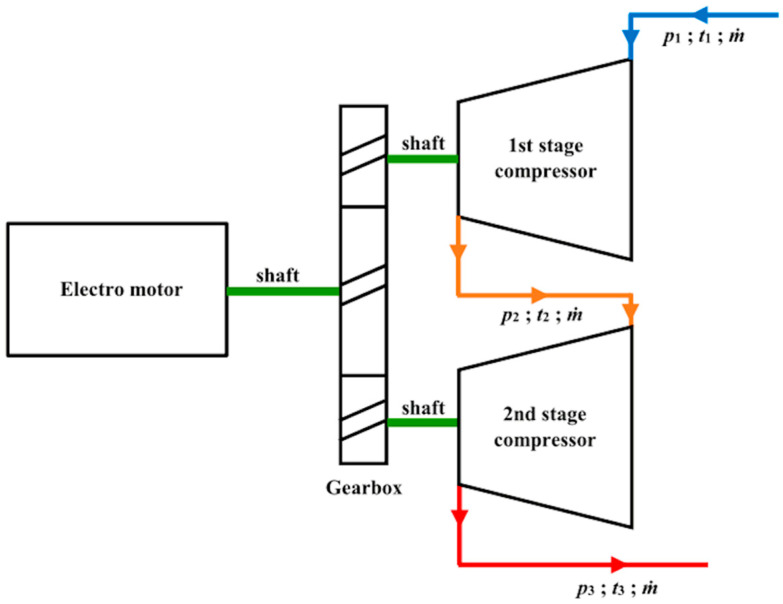
Two-stage compressor outline with given flows.

The schematic outline of the two-stage compressor is given in Figure 1, where *p*_1_, *t*_1_ is LNG vapor pressure and temperature at compressor inlet to the first stage. Delivery pressure and temperature from the compressor first stage and the inlet to the compressor second stage are *p*_2_ and *t*_2_, respectively. Outlet pressure and temperature from the second stage are *p*_3_ and *t*_3_, respectively. The mass flow rate (*ṁ*) is the same through all operation measurement range.

The energy efficiency data for given conditions, provided by the manufacturer are presented in Table 3.

## 3. Measuring Equipment and Measuring Results

Sea trial tests were carried out at different main propulsion shaft rotation speed. The test commenced at the lowest load of the two-stage compressor, where the compressor is delivering all gas to the generator engines, which is at about 46 rpm at the main propulsion shafts. Measuring results (Table 4 and Table 5) were obtained with ship’s standard measuring equipment [34,35,36] for various readings which include pressure, temperature and mass flow rate of LNG vapor at compressor first stage inlet, first stage outlet and second stage outlet. Before commencing the sea trial test, it was required to run diesel generators on the low sulfur diesel oil which is not the preferred option for the charterer due to fuel cost. Once when cooling down temperature is achieved, two-stage compressor received permission to start at higher motor speed. Change over process from diesel oil combustion to gas combustion was carried out according to the producer pre-set sequence. Fuel oil consumption measuring results were carried out during the parallel operation of three and four generators online.

Equipment for electrical readings of voltage, ampere, and power factor is described in [37], and an overview of propulsion shaft revolutions measurements can be found in the literature [38,39]. Measured results are given in Table 5. The power factor of the compressor electromotor varied only for negligible values throughout all measured range at the high voltage control and measuring breaker. Voltage is not changeable in the power control process and is coming from the common ship’s power network, which is 6600 V for this type of vessel. Power network is supplied from four diesel generator units which have the same power output of 7800 kW. For the maneuvering purposes, it is required to have two units to run in parallel, although the power consumption may withstand one unit until dead slow ahead and dead slow astern operation is required. This option is preferred due to safety reasons. Although set voltage should be 6600 V it may be seen from Table 5 that shipyard increased set point for about 2% during the sea trials. The high outlet pressure from the second stage compressor outlet is required due to pressure drops in the gas line to the engines in order to satisfy the engine gas pressure request of 580 kPa. Increasing power at the compressor’s electromotor is achieved by increasing amperage with variable frequency drive [40,41,42]. Measured results with compressor load variations are given in Table 5.

Loaded LNG content (molar ratio) is [43]: CH_4_ (0.9494), C_2_H_6_ (0.0475), C_3_H_8_ (0.0017), *i*-C_4_H_10_ (0.0001), *n*-C_4_H_10_ (0.0001), N_2_ (0.0012).

## 4. Thermodynamic Efficiency Analysis

Two-stage compressor is evaluated for energy efficiency according to recommendations in the following literature [44,45,46,47,48]. Energy efficiency formulation with referring to Figure 1 is given below.

The mass flow rate balance for a general steady-flow system is given as:

(1) $$\sum_{IN}\dot{m}=\sum_{OUT}\dot{m}$$

Energy rate balance for a general steady-flow system is:

(2) $${\dot{Q}}_{IN}+P_{IN}+\sum_{IN}\dot{m}\cdot \left(h+\frac{{\tilde{v}}^{2}}{2}+g\cdot z\right)={\dot{Q}}_{OUT}+P_{OUT}+\sum_{OUT}\dot{m}\cdot \left(h+\frac{{\tilde{v}}^{2}}{2}+g\cdot z\right)$$

In the steady flow processes, the total mass and energy content remains constant in the control volume, thus the total energy rate change of the system is zero:

(3) $${\dot{E}}_{IN}-{\dot{E}}_{OUT}=\frac{d\dot{E}}{dt}=0,$$

and then

(4) $${\dot{E}}_{IN}={\dot{E}}_{OUT}.$$

Energy efficiency is a ratio of useful and used energy streams in the process:

(5) $$\eta_{I}=\frac{{\dot{E}}_{OUT}}{{\dot{E}}_{IN}}=1-\frac{\dot{E}l}{{\dot{E}}_{IN}}.$$

**Figure 2:**
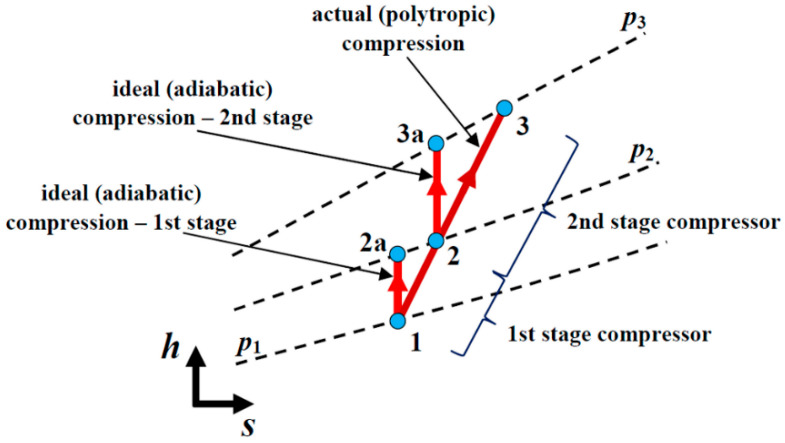
The *h*–*s* diagram of the compression process in the two-stage compressor.

The irreversible adiabatic process is calculated as per equations from (6) to (8) in order to match with the producer’s results given in Table 3. A compression process with ideal or adiabatic compression and the actual (polytropic) process being an irreversible adiabatic process is shown in the h–s diagram in Figure 2.

Energy efficiency of the compressor first stage is:

(6) $$\eta_{I_{1}}=\frac{P_{1adiabatic}}{P_{1actual}}=\frac{\dot{m}\cdot \left(h_{2a}-h_{1}\right)}{\dot{m}\cdot \left(h_{2}-h_{1}\right)}=\frac{\left(h_{2a}-h_{1}\right)}{\left(h_{2}-h_{1}\right)}.$$

Energy efficiency of the compressor second stage is:

(7) $$\eta_{I_{2}}=\frac{P_{2adiabatic}}{P_{2actual}}=\frac{\dot{m}\cdot \left(h_{3a}-h_{2}\right)}{\dot{m}\cdot \left(h_{3}-h_{2}\right)}=\frac{\left(h_{3a}-h_{2}\right)}{\left(h_{3}-h_{2}\right)}.$$

Overall two-stage compressor energy efficiency (according to Figure 2) is:

(8) $$\eta_{I}=\frac{P_{adiabatic}}{P_{actual}}=\frac{\dot{m}\cdot \left(h_{2a}-h_{1}\right)+\dot{m}\cdot \left(h_{3a}-h_{2}\right)}{\dot{m}\cdot \left(h_{3}-h_{1}\right)}=\frac{\left(h_{2a}-h_{1}\right)+\left(h_{3a}-h_{2}\right)}{\left(h_{3}-h_{1}\right)}.$$

Actual measured temperatures, pressures, and mass flow rate are used for calculating the actual (polytropic) power for running the compressor’s first stage only:

(9) $$P_{1actual}=\dot{m}\cdot \left(h_{2}-h_{1}\right),$$

and for running the compressor’s second stage only:

(10) $$P_{2actual}=\dot{m}\cdot \left(h_{3}-h_{2}\right).$$

Mass flow rate at the compressor first stage is:

(11) $$\dot{m}=\frac{P_{1actual}}{\left(h_{2}-h_{1}\right)}.$$

Mass flow rate at the compressor second stage is:

(12) $$\dot{m}=\frac{P_{2actual}}{\left(h_{3}-h_{2}\right)}.$$

Equalling Equations (11) and (12) gives:

(13) $$P_{2actual}=P_{1actual}\cdot \frac{\left(h_{3}-h_{2}\right)}{\left(h_{2}-h_{1}\right)}.$$

The actual power required for running the whole compressor is:

(14) $$P_{actual}=P_{1actual}+P_{2actual}=P_{1actual}+P_{1actual}\cdot \frac{\left(h_{3}-h_{2}\right)}{\left(h_{2}-h_{1}\right)}=P_{1actual}\cdot \left[\frac{\left(h_{3}-h_{2}\right)}{\left(h_{2}-h_{1}\right)}+1\right]=P_{1actual}\cdot \frac{\left(h_{3}-h_{1}\right)}{\left(h_{2}-h_{1}\right)}.$$

For the energy losses, exergy efficiency and exergy destruction calculation the idea is to present all power losses from the electrical power input, which is required to run the compressor electromotor until it’s output. For such analysis, it was required to split the measured electrical power at the common electromotor inlet to the first and second stages of the compressor. The splitting of the power done through the proportion of power at the actual compression irreversible process. Formula for calculation of electric motor power from measured results is drawn from literature [49,50]:

(15) $$P_{EM}=\sqrt{3}\cdot I\cdot U\cdot \cos\varphi .$$

Total electric power for the running of the first and the second compressor stage, which includes all irreversibility’s inside the process such are: electrical losses of the electric motor, mechanical losses inside the power transmission reduction gears, power losses in the shaft seals, etc. equals to:

(16) $$P_{EM}=P_{1EM}+P_{2EM}.$$

Electrical energy distributed to power each compressor stage (first and second) is divided in the same proportions as actual (polytropic) power. Consumed electrical energy for the running of the compressor first stage is then:

(17) $$P_{1EM}=P_{EM}\cdot \frac{\left(h_{2}-h_{1}\right)}{\left(h_{3}-h_{1}\right)}.$$

Consumed electrical energy for the running of the compressor second stage is:

(18) $$P_{2EM}=P_{EM}-P_{1EM}.$$

Energy rate balance of the compressor first stage is:

(19) $$\dot{m}\cdot h_{1}+P_{1EM}=\dot{m}\cdot h_{2}+\dot{E}l_{1}.$$

Energy rate losses of the compressor first stage are:

(20) $$\dot{E}l_{1}=\dot{m}\cdot \left(h_{1}-h_{2}\right)+P_{1EM}.$$

Energy rate balance of the compressor second stage is:

(21) $$\dot{m}\cdot h_{2}+P_{2EM}=\dot{m}\cdot h_{3}+\dot{E}l_{2}.$$

Energy rate losses of the compressor second stage are:

(22) $$\dot{E}l_{2}=\dot{m}\cdot \left(h_{2}-h_{3}\right)+P_{2EM}.$$

Energy rate balance of the two-stage compressor is:

(23) $$\dot{m}\cdot h_{1}+P_{1EM}+\dot{m}\cdot h_{2}+P_{2EM}=\dot{m}\cdot h_{2}+\dot{m}\cdot h_{3}+\dot{E}l.$$

Overall energy rate losses of the two-stage compressor are:

(24) $$\dot{E}l=\dot{m}\cdot \left(h_{1}-h_{3}\right)+P_{EM}.$$

Exergy evaluation of two-stage compressor is performed according to the literature [51,52,53,54,55,56,57,58,59]: exergy rate balance for steady flow systems is:

(25) $$\sum_{IN}\left(\dot{m}\cdot ex\right)+\sum \left(1-\frac{T_{0}}{T}\right)\cdot \dot{Q}=\sum_{OUT}\left(\dot{m}\cdot ex\right)+P+T_{0}\cdot \Delta S,$$

where specific exergy is given by:

(26) $$ex=h-h_{0}-T_{0}\cdot \left(s-s_{0}\right).$$

Exergy rate balance of the compressor first stage is:

(27) $$\dot{m}\cdot ex_{1}+P_{1EM}=\dot{m}\cdot ex_{2}+\dot{E}xd_{1}.$$

Exergy destruction rate of the compressor first stage is:

(28) $$\dot{E}xd_{1}=\dot{m}\cdot \left(ex_{1}-ex_{2}\right)+P_{1EM}.$$

Exergy efficiency of the compressor first stage is:

(29) $$\eta_{{\mathrm{II}}_{1}}=1-\frac{\dot{E}xd_{1}}{\dot{E}x_{IN}}=\frac{\dot{E}x_{OUT}}{\dot{E}x_{IN}}=1-\frac{\dot{m}\cdot ex_{1}-\dot{m}\cdot ex_{2}+P_{1EM}}{P_{1EM}}=\frac{\dot{m}\cdot ex_{2}-\dot{m}\cdot ex_{1}}{P_{1EM}}.$$

Exergy rate balance of the compressor second stage is:

(30) $$\dot{m}\cdot ex_{2}+P_{2EM}=\dot{m}\cdot ex_{3}+\dot{E}xd_{2}.$$

Exergy destruction rate of the compressor second stage is:

(31) $$\dot{E}xd_{2}=\dot{m}\cdot \left(ex_{2}-ex_{3}\right)+P_{2EM}.$$

Exergy efficiency of the compressor second stage is:

(32) $$\eta_{{\mathrm{II}}_{2}}=1-\frac{\dot{E}xd_{2}}{\dot{E}x_{IN}}=\frac{\dot{E}x_{OUT}}{\dot{E}x_{IN}}=1-\frac{\dot{m}\cdot ex_{2}-\dot{m}\cdot ex_{3}+P_{2EM}}{P_{2EM}}=\frac{\dot{m}\cdot ex_{3}-\dot{m}\cdot ex_{2}}{P_{2EM}}.$$

Exergy rate balance of two-stage compressor is:

(33) $$\dot{m}\cdot ex_{1}+P_{1EM}+\dot{m}\cdot ex_{2}+P_{2EM}=\dot{m}\cdot ex_{2}+\dot{m}\cdot ex_{3}+\dot{E}xd.$$

Exergy destruction rate of the two-stage compressor is:

(34) $$\dot{E}xd=\dot{m}\cdot \left(ex_{1}-ex_{3}\right)+P_{EM}.$$

Exergy efficiency of the two-stage compressor is:

(35) $$\eta_{\mathrm{II}}=1-\frac{\dot{E}xd}{\dot{E}x_{IN}}=\frac{\dot{E}x_{OUT}}{\dot{E}x_{IN}}=1-\frac{\dot{m}\cdot ex_{1}-\dot{m}\cdot ex_{3}+P_{EM}}{P_{EM}}=\frac{\dot{m}\cdot ex_{3}-\dot{m}\cdot ex_{1}}{P_{EM}}.$$

The assumed atmospheric condition for exergy calculation is 25 °C and 0.1 MPa according to the literature overview [60,61].

## 5. Energy and Exergy Analysis Results and Discussion

Calculated electromotor power distribution between two compressors are presented in Table 6. As previously mentioned, the LNG carrier has two propulsion shafts and for convenience only average measured rpm of both shafts will be given through the discussion. As can be seen from Table 6, the two-stage compressor is well balanced and load shearing between the two stages is within 0.5%. The first stage is slightly more loaded compared to the second stage. The increase of compressor electromotor power with load variation is about 107 kW. That is a relatively small amount, compared to total power required for a two-stage compressor, which is required for running the unit at the lowest delivery zones.

**Figure 3:**
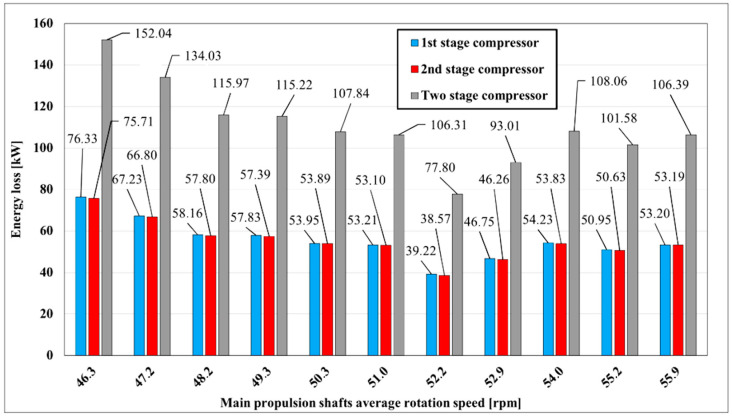
Two-stage compressor energy loss with main propulsion shafts speed variation.

Two-stage compressor energy loss is shown in Figure 3. As the rotation speed of the main propulsion shaft increases, more gas has to be delivered to the generator engines. A higher load at generators is proportional to the withdrawal of more vapor from the cargo tanks. The side effect is that cargo tank pressure is reduced and colder cargo vapor is flowing to the compressor inlet. That process continues and the temperature is going down at rotation speed of main propulsion shafts from 46.3 to 52.2 rpm. Increased mass flow rate and decreased temperature at compressor inlet reduce energy losses and a peak of energy loss reduction is at 52.2 rpm of main propulsion shafts. After 52.2 rpm at main propulsion shafts temperature at compressor’s first stage outlet stabilizes and losses become steady at about 50 kW at each compressor stage, Figure 3. Total energy loss of compressor is higher in the lower running zones and varies from 80 to 150 kW for both compressor stages. Energy losses of the first and the second compressor stages are practically the same through all measured range what is shown in Figure 3.

**Figure 4:**
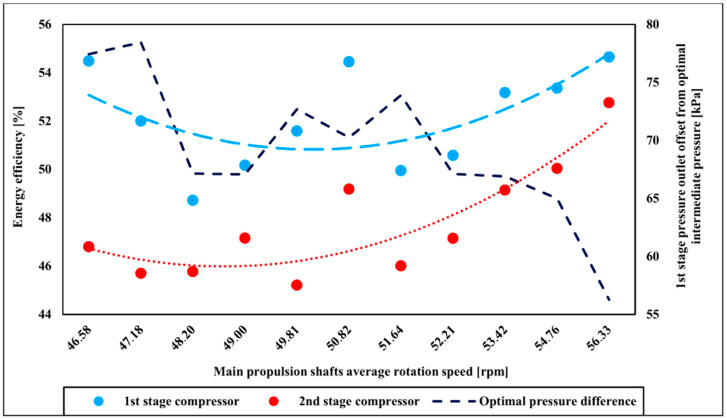
Two-stage compressor energy efficiency with main propulsion shafts speed variation.

The energy efficiency of the compressor is shown in Figure 4. The compressor energy efficiency of the first stage is higher compared to the second stage and is in the average range of about 52%. The energy efficiency of the second stage average range is up to 48%. As compressor increases delivery, energy efficiencies of both stages converge and are for both stages above 50%. This effect is connected to reduced offset from optimal intermediate pressure at the outlet of the first compressor stage [57]. The optimal intermediate pressure is calculated as [57]:

(36) $$p_{2}=\sqrt{p_{1}\cdot p_{3}},$$

where:

*p*_1_—is pressure at the compressor inlet to the first stage [kPa],
*p*_2_—is pressure at the compressor outlet from the first stage [kPa],
*p*_3_—is pressure at the compressor outlet from the second stage [kPa].

The offset is:

(37) $$\Delta p=p_{2}-\sqrt{p_{1}\cdot p_{3}}.$$

As the two-stage compressor mass flow rate becomes higher offset decreases what acts beneficially to the two-stage compressor energy efficiency.

**Figure 5:**
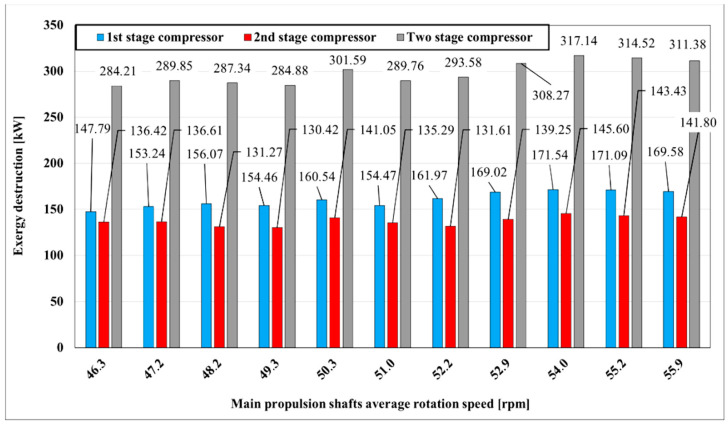
Two-stage compressor exergy destruction with main propulsion shafts speed variation.

The exergy destruction of the two-stage compressor is higher comparing to energy losses where peak destruction is raised to about 320 kW. It may be seen in Figure 5 that the first stage compressor destructs more exergy compared to the second compressor stage in all measured ranges, which was not clearly visible in energy analysis. As the exergy destruction of the first stage compressor is higher compared to the second stage compressor, the efficiency of the first stage compressor will be lower. Destructed exergy of the first stage compressor is from 150 kW at a rotation speed of main propulsion shaft 46.3 rpm and is gradually increased to about 170 kW at 55.9 rpm, whereas destructed exergy of the second stage compressor is almost equally distributed around 140 kW, as shown in Figure 5.

**Figure 6:**
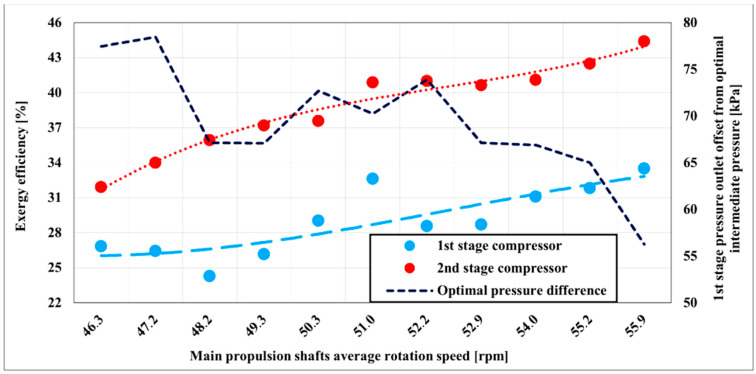
Two-stage compressor exergy efficiency with main propulsion shafts speed variation.

The outcome of exergy destruction analysis is the higher exergy efficiency of the second stage compressor comparing to the first stage compressor in all measured range. The first stage compressor exergy efficiency varies with the compressor load from about 24 to 34% at the measured range. The best running conditions are achieved at a rotation speed of 51 and 55.9 rpm at the main shafts. The second stage of the compressor has a higher exergy efficiency, which varies from about 32 to 45%, but contrary to the first stage of the compressor best results are distributed at a rotation speed of 51 rpm onwards at the main propulsion shafts. By increasing the delivery of the compressor, exergy efficiency increases, however at the same time efficiency offset between two compressors stages becomes higher as well. Optimal intermediate pressure does not have the same effect on exergy efficiency as on energy efficiency. In fact, it is the opposite. As previously explained, also shown in Figure 6, a discrepancy in the exergy efficiency of the first and second stages is about 5% at the beginning, and at the end of the measured range, the discrepancy increases to about 10%. It means that decreasing the optimal intermediate pressure offset exergy efficiency of both compressor stages has better results, but discrepancy in efficiency increases as well. Exergy efficiency is increased smoothly in entire ranges for the second stage of the compressor. Regarding the first stage of the compressor, exergy efficiency is increasing in the whole measured range, but it is not evenly distributed as it is distributed in the second stage.

**Figure 7:**
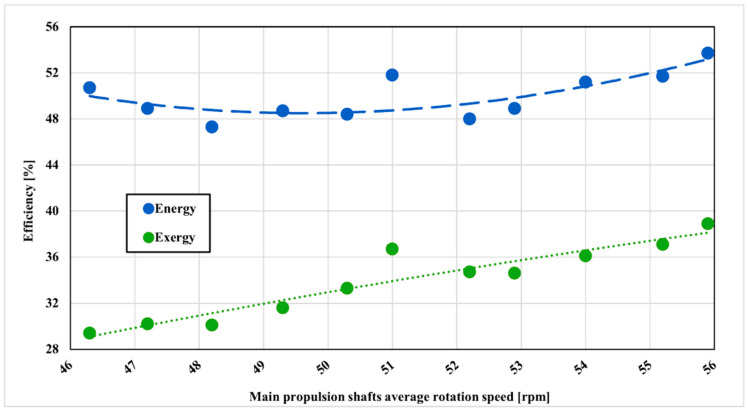
Two-stage compressor energy and exergy efficiency with main propulsion shafts speed variation.

The overall efficiency of the two-stage compressor is given in Figure 7. The energy efficiency of the two-stage compressor in the observed range, in the beginning, is 50.7%. As the rotation speed of the main propulsion shaft increases, energy efficiency is dropping to about 47.3% at 48.2 rpm. The average measured energy efficiency in the whole range is 49.9% and the best energy efficiency is 53.7% at the last measured propulsion shaft rotation speed. Measured results are, on average, about 5% lower compared to the maker’s efficiency. Although calculated efficiency is lower compared to the producer results it has to be noticed that the compressor was running on the lower delivery pressure and higher inlet temperature at the first compressor stage what is affecting compressor efficiency. It could be concluded that calculated efficiency will be even closer to the producer results as running parameters converge to the parameters given by the producer. On the other side, exergy efficiency is lower, about 29% in the beginning and with the increased compressor load overall exergy efficiency increases to about 39%. The average exergy efficiency of the two-stage compressor is 33.9%. As exergy efficiency is taking entropy generation into account it is clearly seen that two-stage cryogenic compressor is not as efficient as it appears through the energy efficiency analysis.

**Figure 8:**
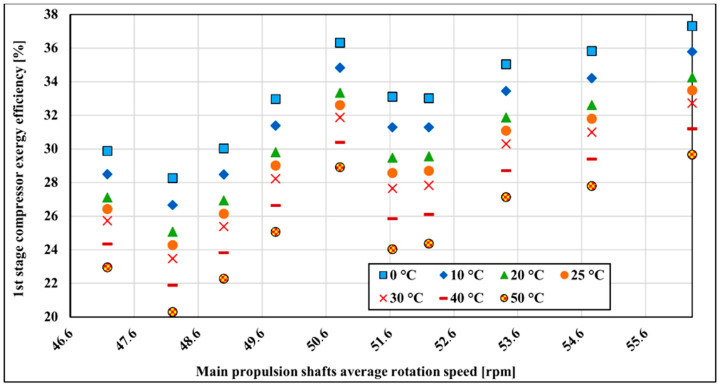
First stage compressor exergy efficiency with surrounding temperature change.

The exergy efficiency of the LNG compressor is influenced by the variation of atmospheric temperature. The change of exergy efficiency in dependence on the outside temperature range from 0 to 50 °C is analyzed. The mentioned temperature range is chosen because these two temperatures are extreme temperatures that the vessel will operate most of the time. The referent temperature for the exergy calculation is 25 °C and 0.1 MPa. Figure 8 shows the first stage compressor’s exergy efficiency changes with temperature variation. As can be seen in Figure 8, higher exergy efficiency is achieved at lower reference temperatures. As temperature increases, exergy efficiency decreases by about 7.7% on average over the whole measured range. The lowest sensitivity to the reference temperature change is in the lower running main propulsion shaft rotation speed where efficiency decreases around 1.18% for every 10 °C from 0 to 50 °C. The highest temperature sensitivity is at 52.83 rpm at the main propulsion shaft rotation speed where it is changing at the rate of 1.81%.

**Figure 9:**
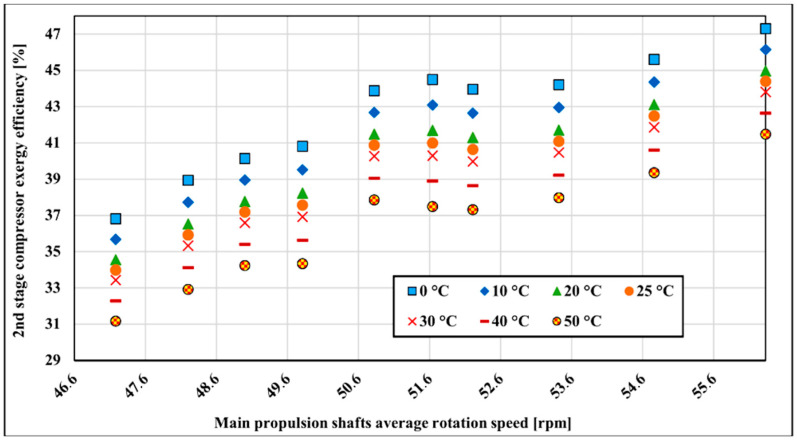
Compressor’s second stage exergy efficiency with surrounding temperature change.

The second compressor stage is less influenced by the surrounding temperature variation compared to the first compressor stage. Exergy changes about 6.1% on average over the measured range (Figure 9). The temperature sensitivity distribution is similar to the first compressor stage and is the lowest in the lower running main propulsion shaft rotation speed where exergy efficiency decreases around 1.01% for every 10 °C from 0–50 °C. The highest temperature sensitivity is at 52.83 rpm of main propulsion shaft rotation speed where it is changing at the rate of 1.39%.

**Figure 10:**
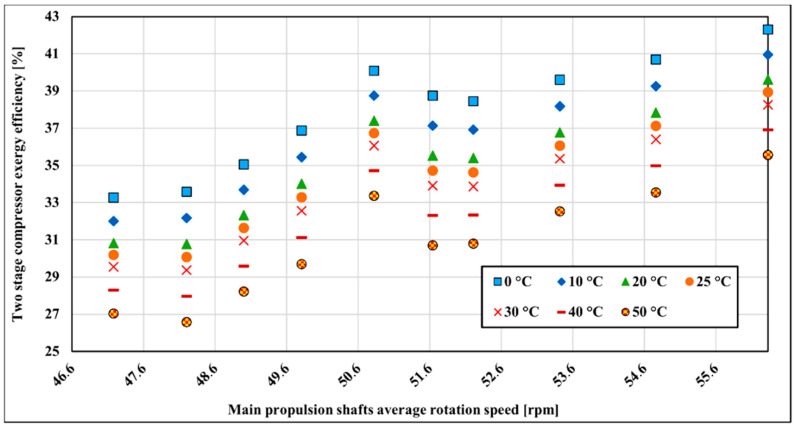
Two-stage compressor exergy efficiency with surrounding temperature change.

Overall compressor exergy efficiency changes are 6.9% on average for the whole measured range with surrounding temperature variations from 0 to 50 °C (Figure 10). The temperature sensitivity distribution is proportional to the compressor first and second stage exergy efficiency change. According to overall results, it is preferable for a compressor to operate at the lowest atmospheric temperatures in order to have better exergy efficiency. However, LNG carriers are sailing all over the world in different climates and ship’s crew and/or compressor operators cannot influence at atmospheric conditions in order to increase the exergy efficiency but this analysis can help in better understanding of compressor processes.

## 6. Variation and Optimization of Temperature at the Compressor’s Second Stage Inlet

In this part, the variation and optimization of temperature at the compressor’s second stage are presented. The proposed new concept of pre-cooling the vapor before the compressor second stage could enable energy saving and increase compressor efficiency.

Part of LNG may be taken from the cargo tank with the purpose to cool pressurized LNG before the compressor’s second stage. Such a scenario will not require forcing vaporizer in the system. When the ship is sailing on the calm sea and boil-off gas is not sufficient in the cargo vapor header part of this vaporized liquid after-cooler will evaporate and maintain sufficient pressure in the cargo tanks. The purpose of forcing vaporizer is to vaporize part of the liquid from the cargo tanks and to maintain compressor suction pressure in the allowed ranges and to avoid reducing cargo tank pressure excessively. If cooler is installed between two stages of compressor this could partially replace the forcing vaporizer. Cooling between two stages may be achieved either with additional cooler or with direct injection of part of the liquid inside the outlet of the first stage compressor by the spray pump.

**Figure 11:**
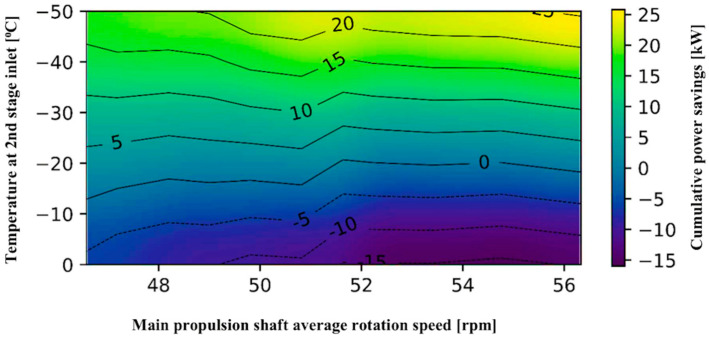
Two-stage compressor cumulative power optimum saving with second stage inlet temperature variation.

Actual (polytropic) power saving depends on the compressor’s second stage inlet temperature is given in Figure 11. The temperature range from 0 to −50 °C is chosen. The energy efficiency of the two-stage compressor had been taken from the previous calculation for the various purposes and is set as the fixed value. The cumulative calculated power needed for increasing pressure to measured set pressure of 650 kPa at different loads is compared to actual measured power by changing inlet temperatures of the second stage. Running off the main propulsion shaft from 46.5 to 56.3 rpm is giving a cumulative power saving of two-stage compressor from 7 to 25.8 kW in the temperature range from –30 to −50 °C at compressor inlet to the second stage. For the pre-cooling in the range from –20 to −30 °C at compressor inlet to the second stage cumulative power savings are in the range from 0.3 to 10 kW but from 51.64 to 54.76 rpm at −20 °C at compressor inlet to the second stage there is no cumulative power savings. Temperatures above −20 °C at compressor inlet to the second stage give negative results and the compressor is consuming more power in such a working regime in all running zones.

## 7. Conclusions

In this paper, the energy and exergy evaluation of the two-stage axial vapor compressor is presented. Data from cryogenic compressors on the dual-fuel diesel–electric LNG carrier were measured and analyzed to investigate compressor energy and exergy efficiency in real exploitation conditions. Operating parameters of this type of compressor, especially during on-board performance are rarely investigated, so the presented analysis enables insight into better understanding the compressor processes in different operating regimes. The results show how main engine load variation has an influence on compressor performance which is not found in the producers’ manuals or scientific literature.

Energy efficiency of compressor first and second stages has the highest value at the lowest main engine rotation speed and at the highest delivery ranges. The presented results show that the overall average calculated energy efficiency of the two-stage compressor is 49.9% and the best energy efficiency is about 54% at the last measured propulsion shaft rotation speed. At the average rotation speed of the main propulsion shaft of 48.2 rpm, energy efficiency decreases to the lowest value of about 47%. Calculated efficiency is lower compared to the producer results but it has to be noticed that the compressor was running on the lower delivery pressure and higher inlet temperature at the first compressor stage what is affecting compressor efficiency. Producers usually present only the best results and at full compressor capacity but in this paper, the whole range of compressor regime was analyzed. Although the compressor first and second stages are well balanced, the first stage will achieve higher efficiency if the pressure rise offset is lower.

Compressor exergy efficiency is lower, about 29% in the beginning and with the increasing compressor load overall exergy efficiency increases to about 39%. The average exergy efficiency of the two-stage compressor is 33.9%. However, compressor manufacturers never evaluate the exergy efficiency of the compressor as its efficiency is lower compared to energy efficiency. According to exergy analysis, the two-stage compressor is not very efficient equipment and there is still room for design improvements.

Examination of the exergy efficiency in dependence on surrounding temperature shows that the two-stage compressor is more effective at lower temperatures. In that respect, the two-stage compressor is behaving similarly to the steam turbines [61,62]. The compressor first stage is more sensitive to temperature changes as it works with higher temperature differences than the second stage. Overall compressor exergy efficiency changes are 6.9% on average for the whole measured range with surrounding temperature variations from 0 to 50 °C. Temperature sensitivity analysis shows that the lowest compressor first stage exergy efficiency decrease is around 1.18% with every 10 °C from 0 to 50 °C at the lowest measured main propulsion shaft average rotation speed. The highest temperature sensitivity is at 52.83 rpm at the main propulsion shaft rotation speed where it is changing at the rate of 1.81%. The lowest second compressor stage exergy efficiency change is at lowest measured main propulsion average speed around 1.01% with every 10 °C from 0 to 50 °C and the highest temperature sensitivity is at 52.83 rpm of main propulsion shaft rotation speed where it is changing at the rate of 1.39%.

The proposed new concept of inter-cooling of the LNG after the first stage of the compressor could increase the efficiency of the compressor and reduce consumed power at electromotor. In the lower running zone of the main propulsion, shafts total consumed power at electromotor will be 7.85% less than real operational power. In the upper running zone of the main propeller shafts, this saving decreases by about 1% and is 6.84%.

The results of this analysis could be useful for a broad audience and for ship owners, compressor operators, and producers.

## Figures and Tables

**Table 1:** Overview of compressor mass flow rate, working medium and assumed efficiencies.

Pressure	Medium	Mass Flow Rate	Simulation	Type of Compressor	Efficiency	Source
[MPa]	-	[kg/s]	-	-	η_I_ or η_II_ [%]	-
<1	LNG	1.39	HYSIS	Unknown	85−96	[15]
2.4	Nitrogen	1.7	Aspen	Unknown	81.98	[16]
0.55–2.5	Propane and LNG	0.9–3.44 [mol/s]	Analysis	Unknown	66−79	[17]
0.2076	Dry air	0.3134	EES	Unknown	62.7	[18]
1.305	Methane/Ethylene/Propane	3.95	HYSIS	Unknown	75	[19]
1.373	Ethane/Propane/Isobutene	2.52			85	

**Table 2:** Two stage compressor main design particulars.

Two Stage Compressor Main Characteristics	Operation Parameter
Capacity at full speed	3200 m^3^/h
Capacity at half speed	520 m^3^/h
Suction pressure	104 kPa
Suction temperature	−120 °C
Discharge pressure at full speed	700 kPa
Discharge pressure at half speed	160 kPa
Shaft rpm at full speed	29,775 rpm
Shaft rpm at half speed	14,888 rpm
Electric motor power	610 kW

**Table 3:** Two-stage compressor design operating parameters [33].

Parameter	Unit	1	2	3	4
Gas		CH_4_	90CH_4_/10N_2_	BOG	Half speed
Inlet pressure	[kPa]	104	104	104	104
Outlet pressure	[kPa]	700	700	700	700
Inlet temperature	[°C]	−120	−120	−120	−120
Outlet temperature	[°C]	41.5	55.4	42.7	−75.9
Energy efficiency	[%]	55	52.1	55.2	39
Coupling power	[kW]	485	514	488	68

**Table 4:** Two-stage compressor measured pressure and temperatures with main propulsion shafts speed variations.

Port Side Shaft Output	Starboard Side Shaft Output	First Stage Inlet Pressure	First Stage Inlet Temperature	First Stage Outlet Pressure	First Stage Outlet Temperature	Second Stage Outlet Pressure	Second Stage Outlet Temperature
[rpm]	[rpm]	[kPa]	[°C]	[kPa]	[°C]	[kPa]	[°C]
46.05	46.58	112.5	−109.2	355	−13	684.7	75.2
47.16	47.18	112.2	−113.9	357.8	−15	695.5	75.8
48.17	48.2	111.8	−115.9	340	−16.8	665.9	74.5
49.56	49	111.5	−114.3	341.4	−16.2	674.9	74
50.88	49.81	109.9	−113.8	341.3	−16.6	656.5	73.3
51.21	50.82	109.7	−110.8	345	−15.7	688.1	72.3
52.83	51.64	109.9	−119	347.8	−20.6	682.6	69.6
53.61	52.21	109.1	−116.5	335.7	−20.1	661.2	68.7
54.54	53.42	108.3	−114	336.6	−19.6	671.6	67.6
55.63	54.76	108	−114	335	−20.1	675	66.8
55.37	56.33	106.8	−109.6	321.5	−18.2	658.7	67

**Table 5:** Two-stage compressor measured mass flow rate, voltage, amperage and power factor with main propulsion shafts speed variations.

Port Side Shaft Output	Starboard Side Shaft Output	Mass Flow Rate	Voltage	Amperage	Measured Power Factor
[rpm]	[rpm]	[kg/h]	[V]	[A]	[φ]
46.05	46.58	2320	6764	40.4	0.85
47.16	47.18	2535	6763	41.7	0.85
48.17	48.2	2650	6759	41.3	0.85
49.56	49	2741	6756	41.9	0.85
50.88	49.81	3150	6764	45.4	0.85
51.21	50.82	3291	6763	46	0.85
52.83	51.64	3382	6759	45.2	0.85
53.61	52.21	3509	6758	47.4	0.85
54.54	53.42	3672	6752	49.9	0.85
55.63	54.76	3792	6755	50.3	0.85
55.37	56.33	3926	6752	51.3	0.85

**Table 6:** Two-stage compressor measured electromotor power and calculated load shearing ratio between two compressors.

Main Engines Shaft Output	First Stage Calculated Power	Second Stage Calculated Power	Measured Electromotor Power	First Stage Load Share	Second Stage Load Share
[rpm]	[kW]	[kW]	[kW]	[%]	[%]
46.32	201.99	200.33	402.31	50.21	49.79
47.17	208.27	206.93	415.20	50.16	49.84
48.19	206.12	204.85	410.97	50.15	49.85
49.28	209.17	207.59	416.76	50.19	49.81
50.35	226.18	225.92	425.10	50.03	49.97
51.02	229.24	228.77	458.01	50.05	49.95
52.24	226.76	223.02	449.78	50.42	49.58
52.91	237.06	234.54	471.60	50.27	49.73
53.98	248.94	247.10	496.04	50.19	49.81
55.20	250.92	249.32	500.23	50.16	49.84
55.85	254.98	254.97	509.95	50.00	50.00

## Nomenclature

**Latin Symbols**	
*Ėl*	energy rate loss, kW
*ex*	specific exergy, kJ/kg
*Ėxd*	exergy destruction rate, kW
*g*	gravitational acceleration, m/s^2^
*h*	specific enthalpy, kJ/kg
*I*	amperage, A
*ṁ*	mass flow rate, kg/h
*P*	power, kW
$\dot{Q}$	heat flow rate, kW
$\dot{S}$	entropy rate, kW/K
*T*	temperature, K
*U*	voltage, V
$\tilde{v}$	speed, m/s
*z*	height, m
**Greek symbols**	
*η_I_*	energy efficiency, %
*η_II_*	exergy efficiency, %
*φ*	power factor
**Abbreviations**	
BOG	boil off gas
DFDE	dual fuel diesel electric
EM	electromotor
LNG	liquefied natural gas
rpm	revolutions per minute
**Subscript**	
a	adiabatic
